# A Bayesian model to analyse the association of comorbidities with biosimilar treatment retention in a non-medical switch scenario in patients with inflammatory rheumatic musculoskeletal diseases

**DOI:** 10.1186/s13075-024-03386-7

**Published:** 2024-09-04

**Authors:** Imke Redeker, Stefan Moustakis, Styliani Tsiami, Xenofon Baraliakos, David Kiefer, Ioana Andreica, Björn Buehring, Jürgen Braun, Uta Kiltz

**Affiliations:** 1 Ruhr-Universität Bochum, Bochum, Germany; 2 Rheumazentrum Ruhrgebiet, Ruhr-Universität Bochum, Claudiusstrasse 45, Herne, 44649 Germany; 3 Krankenhaus St. Josef, Wuppertal, Germany; 4 RVZ Steglitz, Berlin, Germany

**Keywords:** Biosimilars, Adherence, Comorbidities

## Abstract

**Objectives:**

To analyse clinical outcomes of a non-medical switch from originator adalimumab (ADA) to its ABP501 biosimilar (ABP) over 6 months in patients with inflammatory rheumatic musculoskeletal diseases (RMD) in relation to comorbidity as a risk factor for therapy discontinuation.

**Methods:**

RMD patients switching from originator ADA to ABP were identified from a large routine database from October 2018 onwards. Documented clinical data at the time of non-medical switching (baseline), and at 3 and 6 months were collected. Comorbidities were represented by the Charlson Comorbidity Index (CCI) at baseline and patients were categorized based on CCI > 0. Differences in the ABP retention rate over 6 months between patients with CCI = 0 and patients with CCI > 0 were analysed using Bayesian exponential regression.

**Results:**

A total of 111 patients with axial spondyloarthritis (*n* = 68), rheumatoid arthritis (*n* = 23) and psoriatic arthritis (*n* = 15), were identified, 74.8% of whom had continued treatment with ABP after 6 months, while a smaller proportion had either switched to another ADA biosimilar (10.8%), switched back to originator ADA (7.2%), switched to a different biologic (3.6%), or dropped out (3.6%). At baseline, a CCI > 0 was found in 38% of patients. Cardiovascular comorbidities (40%) were most prevalent followed by diseases of the skin (33%), the gastrointestinal tract (20%) and the eye (20%). ABP treatment was continued after 6 months in 74% of patients with CCI = 0 and in 76% with CCI > 0. Bayesian analysis showed only a small difference (months) in the APB continuation rate between groups (estimate 0.0012, 95% credible interval (CrI) -0.0337 to 0.0361). Adjusting for age, sex, and disease subtype revealed somewhat shorter retention rates for patients with CCI > 0, but the distribution of the difference included 0 (estimate -0.0689, 95% CrI -0.2246 to 0.0234).

**Conclusion:**

In a non-medical switch scenario of RMD patients, there was no evidence for a considerable difference in ABP retention rates over 6 months between comorbidity groups.

**Supplementary Information:**

The online version contains supplementary material available at 10.1186/s13075-024-03386-7.

## Introduction

In patients with inflammatory rheumatic musculoskeletal diseases (RMD) treated with biologic disease modifying drugs (bDMARDs) such as adalimumab (ADA) retention rates may be impaired due to loss of efficacy—for example due to antidrug antibody formation or other mechanisms. Thus, about 50% of patients were reported to discontinue ADA within a period of 5 years [1]. With the introduction of ADA biosimilar DMARDs (bsDMARDs) in October 2018, non-medical switching, e.g. switching for economic reasons, from originator ADA to bsDMARD ADA has rapidly become part of daily practice in rheumatologic care in Germany [2].

Indeed, the availability and of course the prize of bsDMARDs has definitely encouraged switching to products with similar efficacy but lower prices. A lot of data on the efficacy and safety of bsDMARDs, also in routine care, have been published in the last years [3]. A relatively high retention rate after switching from originator etanercept to its bsDMARD has recently been reported by our group, and we found no evidence for a relevant nocebo effect [3]. However, there were also other studies with different results, and the reasons for treatment discontinuation are still poorly understood [4].

Comorbidity has recently gained much attention in patients with RMD, since it may not only increase morbidity and mortality of RMD patients, especially in chronic inflammatory states, but also confound disease activity and limit drug intake [5]. Comorbidity may influence the health and disease status of patients, and this may also have an impact on treatment decisions. The presence of comorbidities has indeed been shown to be associated with disease activity, functional impairment, health-related quality of life and work productivity of patients with axial spondyloarthritis (axSpA), psoriatic arthritis (PsA) and rheumatoid arthritis (RA) [6–8]. In a recent analysis of claims data in Germany, the number of comorbidities was significantly associated with disease activity, tender/swollen joint counts and physical function [7, 9]. Data from the British Society for Rheumatology Biologics Register in Ankylosing Spondylitis (BSRBR-AS) showed that a higher number of comorbidities was associated with an increase in disease activity and spinal pain [10]. Moreover, data from the BSRBR-AS showed poorer treatment outcomes of patients with axSpA who had comorbidities and shorter retention rates of bDMARDs in patients with PsA who had a Charlson Comorbidity Index (CCI) ≥ 2 [8, 10]. Especially, treatment discontinuation was more often observed in patients with 2 (hazard ratio (HR) 1.3) and ≥ 3 comorbidities (HR 2.2) compared to none [10]. Predictors of discontinuation of first bDMARD include comorbidities, smoking, and worse overall health in patients with RA [11]. Data on bsDMARDs retention in patients with and without comorbidities are scarce.

### Objectives

The aim of this study was to analyse clinical outcomes of a systematic non-medical switch from originator ADA to ADA ABP501 bsDMARD (ABP) over 6 months in patients with inflammatory RMD in relation to comorbidity as a potential risk factor for treatment discontinuation.

## Methods

### Study design

A retrospective chart review of patients with inflammatory RMD who had been treated with originator ADA in daily routine in our tertiary center in October 2018 was performed. The Ethical Committee of the Ruhr-Universität Bochum, Germany, approved the study (reference number 20–7066-BR).

### Study population

Clinical data of all adult patients who had been treated with originator ADA in October 2018 and who had been switched to bsDMARD ABP subsequently for non-medical reasons thereafter were identified in our hospital information system with a large database of structured routine clinical records. Retrospective data from the 6 month-period following the non-medical switch were analysed.

### Clinical data

Patients and disease characteristics documented at baseline (the time of switching) and at 3 and 6 months were compared. The information comprised age, gender, disease subtype (axSpA, RA, PsA), comorbidities, C-reactive protein (CRP), disease activity, physical function, treatment, and adverse events. The presence of comorbidities was defined in terms of a positive Charlson comorbidity index (CCI) which takes relevant comorbidities into account that were chosen based on their adjusted risk of mortality [12]. The axSpA concerning extra-musculoskeletal manifestations inflammatory bowel disease (IBD), skin psoriasis and uveitis are not part of the CCI definition. Disease activity was assessed in RA and PsA patients with the 28-joint Disease Activity Score (DAS28) and in axSpA patients with the Bath Ankylosing Spondylitis (AS) Disease Activity Index (BASDAI) and the AS Disease Activity Score (ASDAS) [13–15]. Physical function in RA and PsA patients was assessed by the Funktionsfragebogen Hannover (FFbH), which strongly correlates with the Health Assessment Questionnaire (HAQ) [16]. Values of FFbH were converted into HAQ values by the published formula: HAQ score = 3.16 − (0.028 × FFbH score) [16]. Physical function was assessed in axSpA patients by using the Bath AS Functional Index (BASFI) [17]. Information on relevant clinical outcomes of treatment with the bsDMARD ABP, including reasons for discontinuation, switch to other mode of actions or back-switch to originator ADA as well as reports about adverse events, were extracted for each visit. All available patient contacts between last administration of the originator bDMARD ADA from the 4th quarter of 2018 to the 3rd quarter of 2019 were analysed. Follow-up visits occurred approximately every 12 weeks up to 6 months.

### Statistics

Descriptive data are presented with means and standard deviations (SD) or the median and interquartile range (IQR) when referring to continuous variables and as absolute frequencies and percentages (%) when referring to categorical ones. For each disease subtype, scores for disease activity and physical function at baseline stratified by CCI > 0 were calculated. The association between ABP retention rates after 6 months and comorbidities, represented by CCI > 0, was analysed using a Bayesian exponential regression model adjusted for age, sex, and disease subtype.

A Bayesian approach was preferred over a frequentist approach to obtain full posterior information instead of point-estimates only. Normal distributions with a mean of zero and a standard deviation of 1 were used as prior distributions for all model parameters.

Summarised results are presented as mean with 95% credible intervals (CrI).

Data analyses were performed with R 4.3.2, including the package brms.

## Results

A total of 121 RMD patients on treatment with originator ADA were switched to the bsDMARD ABP, of which 111 had non-missing value for CCI and were included in the analysis. 68 of which had axSpA, 23 RA, and 15 PsA. The remaining 5 patients had other diseases, including juvenile idiopathic arthritis and synovitis-acne-pustulosis-hyperostosis-osteitis syndrome. Patients’ disease characteristics at baseline stratified by disease subtype are shown in Table 1 with disease activity and physical function depicted in supplementary Fig. 1. Patients with axSpA were younger than RA and PsA patients. More than half of patients with RA (61.5%) or axSpA (56.0%) presented with increased disease activity (DAS28 ≥ 3.2 or ASDAS ≥ 2.1) at baseline, while only 16.7% of patients with PsA had active disease (DAS28 ≥ 3.2). At baseline, the average ASDAS was 2.2 (1.1) in axSpA patients, and the average DAS28 was 3.3 (0.9) and 2.4 (0.9) in RA and PsA patients, respectively. Physical function, assessed by HAQ, was impaired in RA (1.4 (0.8)) and PsA (1.0 (0.7)), and, using BASFI, also in axSpA (3.5 (2.5)). Intake of csDMARDs was higher in patients with RA (70%) or PsA (53%) than in axSpA patients (25%), while glucocorticoid intake was also increased in RA patients (Table 1).

**Fig. 1:**
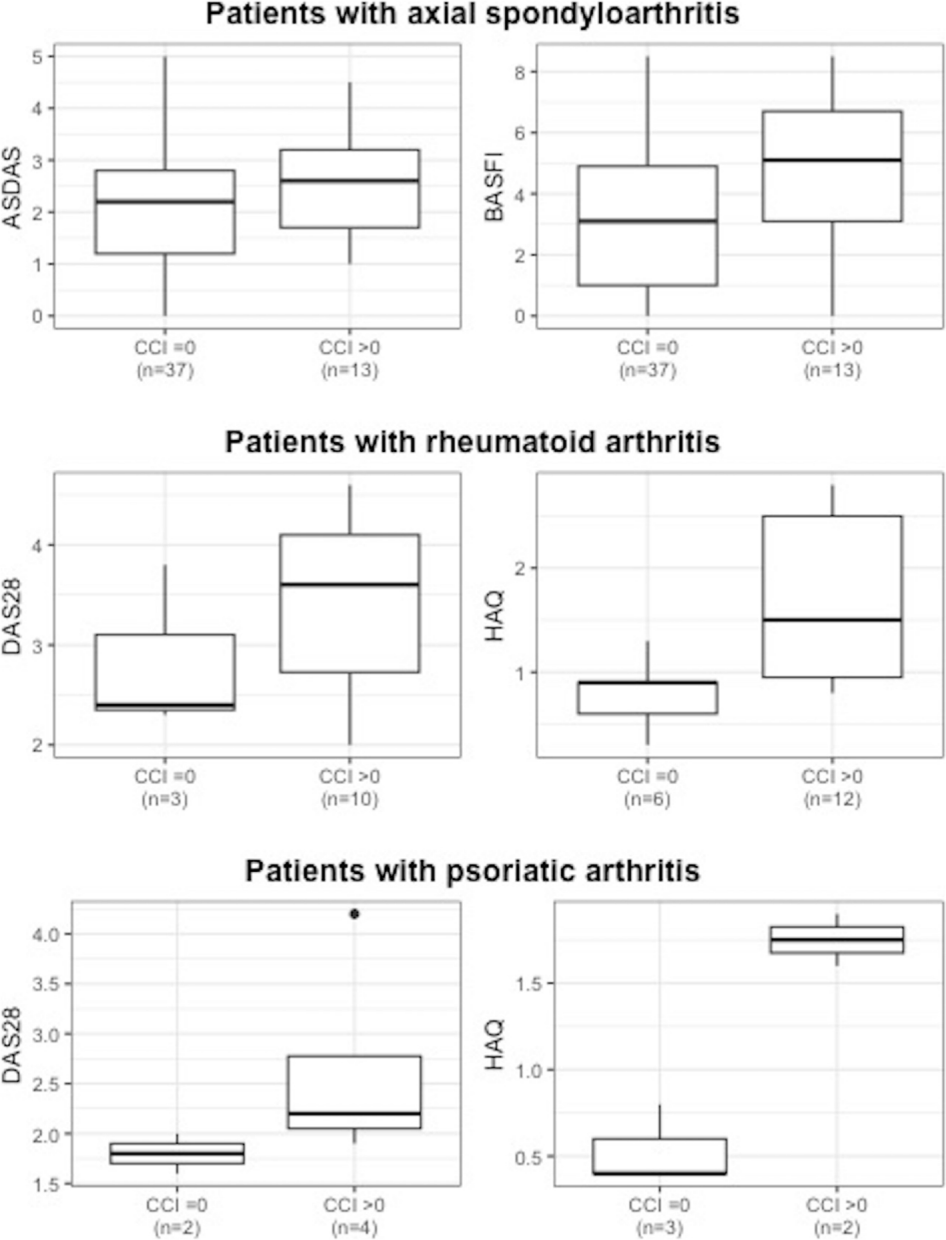
Disease activity and physical function at baseline stratified by Charlson Comorbidity Index. ASDAS: Ankylosing Spondylitis Disease Activity Score; BASFI: Bath Ankylosing Spondylitis Functional Index; CCI: Charlson Comorbidity Index; DAS28: 28-joint Disease Activity Score; HAQ: Health Assessment Questionnaire

**Table 1:** Patients and disease characteristics stratified by disease subtype

**Characteristic**	**N**	**Diagnosis**			
		**axSpA** ***N* = 68**^a^	**RA** ***N* = 23**^a^	**PsA** ***N* = 15**^a^	**Other** ***N* = 5**^a^
Age (years)	111	47 (13)	65 (12)	51 (11)	42 (14)
Male	111	46 (67.6%)	9 (39.1%)	7 (46.7%)	3 (60.0%)
Disease duration (years)	111	5.4 (5.0)	5.4 (3.4)	8.5 (10.1)	12.2 (16.2)
CRP, mg/dl, median (IQR)	110	0.20 (0.10, 0.30)	0.20 (0.10, 0.25)	0.20 (0.10, 0.30)	0.05 (0.00, 0.13)
Outcome after 6 months	111				
Continuation bsDMARD ABP		52 (76.5%)	17 (73.9%)	11 (73.3%)	3 (60.0%)
Switch to other bsDMARD ADA		8 (11.8%)	1 (4.3%)	2 (13.3%)	1 (20.0%)
Back-switch to originator ADA		3 (4.4%)	2 (8.7%)	2 (13.3%)	1 (20.0%)
Switch to other bDMARD		3 (4.4%)	1 (4.3%)	0 (0.0%)	0 (0.0%)
Drop out		2 (2.9%)	2 (8.7%)	0 (0.0%)	0 (0.0%)
Originator ADA was first bDMARD	111	43 (63.2%)	13 (56.5%)	8 (53.3%)	4 (80.0%)
Duration originator ADA therapy (month)	111	39 (27)	44 (29)	35 (29)	61 (28)
Number of previous csDMARD therapies	111				
0		36 (52.9%)	1 (4.3%)	2 (13.3%)	1 (20.0%)
1		23 (33.8%)	7 (30.4%)	8 (53.3%)	1 (20.0%)
≥2		9 (13.2%)	15 (65.2%)	5 (33.3%)	3 (60.0%)
Number of previous bDMARD therapies	111				
1		44 (64.7%)	13 (56.5%)	8 (53.3%)	4 (80.0%)
≥2		24 (35.3%)	10 (43.5%)	7 (46.7%)	1 (20.0%)
Current csDMARDs intake	111	17 (25.0%)	16 (69.6%)	8 (53.3%)	2 (40.0%)
Current NSAIDs intake	111	11 (16.2%)	2 (8.7%)	3 (20.0%)	0 (0.0%)
Current glucocorticoids intake	110				
none		61 (91.0%)	13 (56.5%)	14 (93.3%)	4 (80.0%)
<5 mg		2 (3.0%)	7 (30.4%)	0 (0.0%)	1 (20.0%)
5-10mg		4 (6.0%)	3 (13.0%)	1 (6.7%)	0 (0.0%)
Charlson Comorbidity Index	111				
0		48 (70.6%)	8 (34.8%)	9 (60.0%)	4 (80.0%)
1		11 (16.2%)	6 (26.1%)	3 (20.0%)	1 (20.0%)
≥2		9 (13.2%)	9 (39.1%)	3 (20.0%)	0 (0.0%)
Gastroenterological comorbidities	111	15 (22.1%)	6 (26.1%)	1 (6.7%)	0 (0.0%)
Inflammatory bowel disease		6 (8.8%)	1 (4.3%)	0 (0%)	0 (0%)
Hepatic comorbidities	111	2 (2.9%)	4 (17.4%)	2 (13.3%)	0 (0.0%)
Hematological conditions	111	2 (2.9%)	2 (8.7%)	2 (13.3%)	0 (0.0%)
Cardiovascular comorbidities	111	22 (32.4%)	14 (60.9%)	5 (33.3%)	3 (60.0%)
Neurological and psychological comorbidities	111	12 (17.6%)	2 (8.7%)	5 (33.3%)	0 (0.0%)
Metabolic comorbidities	111	5 (7.4%)	5 (21.7%)	4 (26.7%)	2 (40.0%)
Osteoporosis	111	8 (11.8%)	10 (43.5%)	1 (6.7%)	1 (20.0%)
Lung diseases	111	6 (8.8%)	5 (21.7%)	0 (0.0%)	2 (40.0%)
Skin diseases	111	18 (26.5%)	6 (26.1%)	12 (80.0%)	1 (20.0%)
Skin psoriasis		9 (13%)	2 (8.7%)	9 (60%)	1 (20%)
Eye diseases	111	16 (23.5%)	2 (8.7%)	1 (6.7%)	3 (60.0%)
Uveitis		5 (7.4%)	1 (4.3%)	1 (6.7%)	3 (60%)
Kidney diseases	111	7 (10.3%)	3 (13.0%)	0 (0.0%)	2 (40.0%)

*ABP* ABP501 biosimilar, *ADA* adalimumab, *axSpA* axial spondyloarthritis, *b/bs/csDMARD(s)* biological/biosimilar/conventional synthetic disease-modifying anti-rheumatic drug(s), *CRP* C-reactive protein, *IQR* interquartile range, *NSAID(s)* nonsteroidal anti-inflammatory drug(s), *PsA* psoriasis arthritis, *RA* rheumatoid arthritis, *SD* standard deviation

^a^If not stated otherwise, data are reported as mean (SD) and n (%)

Over 60% of patients had a CCI score of zero, though there were differences between disease subtypes. RA patients were comparatively older (mean age 65 years) and had the highest mean CCI score (1.8) (Table 1). The most prevalent comorbidities were cardiovascular comorbidities (40%, with arterial hypertension as the most prevalent in 34 patients) followed by skin diseases (33%, with skin psoriasis in 21 patients), gastroenterological comorbidities (20%, with gastroesophageal reflux and IBD in 7 patients each and 6 patients with gastritis) and eye diseases (20%, with uveitis in 10 patients and 10 patients with a variety of different non-inflammatory eye diseases). Of note, IBD was present in 6 axSpA patients out of 7 patients with IBD in total, skin psoriasis was present in 9 PsA and 9 axSpA patients out 21 patients with skin psoriasis in total, and uveitis was present in 5 axSpA patients out of 10 patients with uveitis in total. Patients with a CCI score > 0 tended to have poorer disease activity and physical function than patients with a CCI score of zero (Fig. 1).

**Fig. 2:**
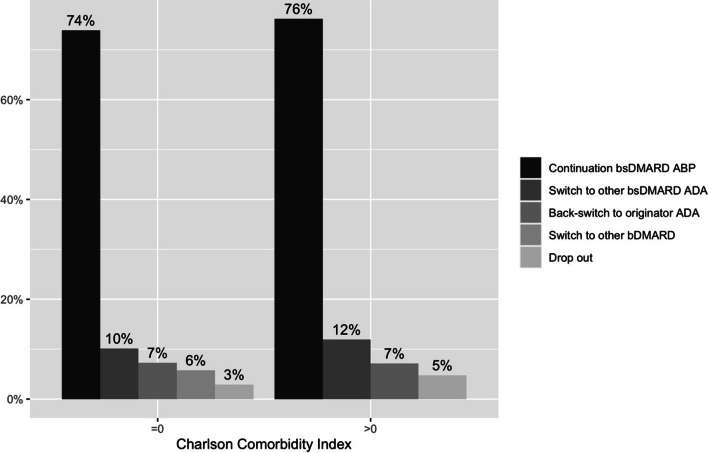
Treatment retention after 6 months stratified by Charlson Comorbidity Index. ABP: ABP501 biosimilar; ADA: adalimumab; b/bsDMARD: biological / biosimilar disease-modifying anti-rheumatic drug

About three-quarters (74.8%) of patients (*N* = 83) continued treatment with the bsDMARD ABP after 6 months, while a small proportion either switched to another ADA bsDMARD (10.8%, *N* = 12), switched back to originator ADA (7.2%, *N* = 8), switched to a different bDMARD (3.6%, *N* = 4), or dropped out (3.6%, *N* = 4). Discontinuation of ABP occurred as early as week 4 with a mean of 14 weeks. The main reason for these switches was the occurrence of adverse events. These were mostly subjective complaints, of which pain was the most frequent. Patients and disease characteristics at baseline stratified by the outcomes after 6 months are depicted in supplementary Table 1. Patients who continued ABP treatment were, on average, older (53 years) and had a longer disease duration (6.8 years) than those who switched to another treatment (supplementary Table 1). Moreover, before the non-medical switch to ABP, they had a longer treatment duration with originator ADA (44 months on average). This was only topped by those who switched back to originator ADA (49 months on average). Treatment retention after 6 months varied only slightly between patients with a CCI score of zero and those with a higher CCI score (Fig. 2).

**Fig. 3:**
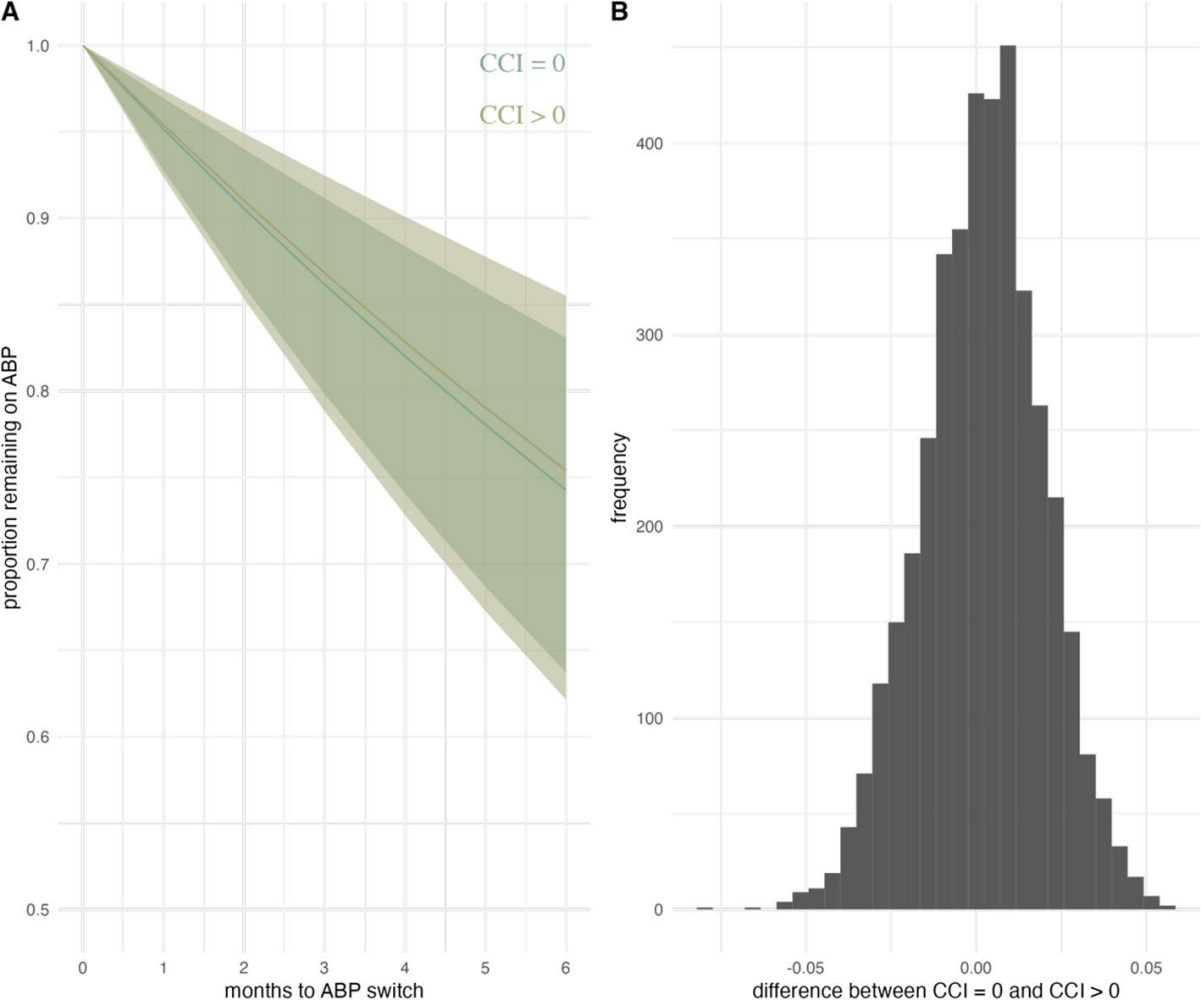
Probability of continuing ABP stratified by CCI (**A**) and posterior distribution of difference in ABP retention between CCI groups (**B**) in patients with RMD over six months estimated using Bayesian exponential regression. ABP: ABP501 biosimilar; CCI: Charlson Comorbidity Index; RMD: inflammatory rheumatic musculoskeletal diseases

Bayesian analysis showed that the estimated difference in APB continuation rate between groups (in months) was small (estimate 0.0012, 95% CrI -0.0337 to 0.0361). The distribution of the estimated difference is depicted in Fig. 3.

**Fig. 4:**
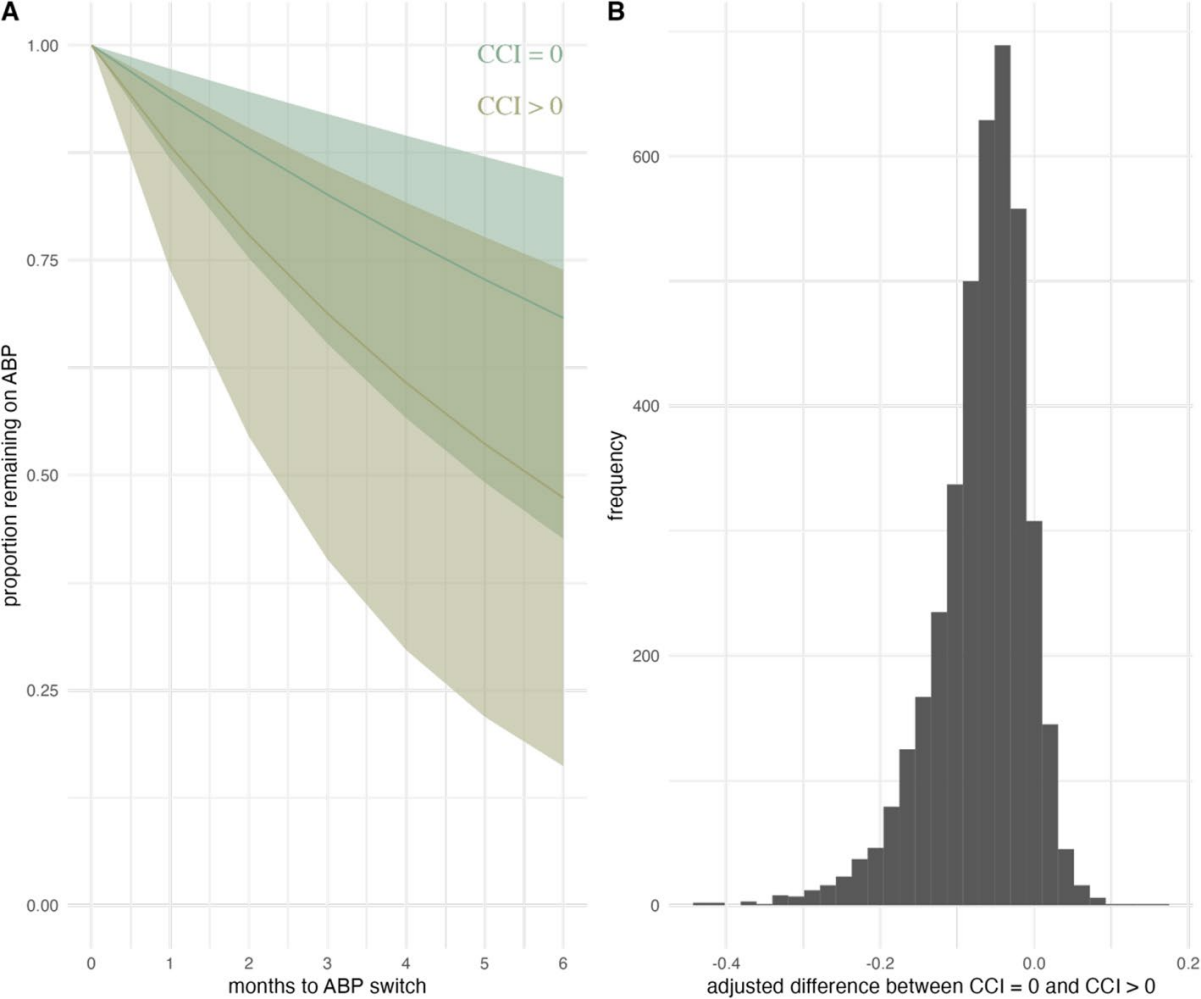
Probability of continuing ABP stratified by CCI (**A**) and posterior distribution of difference in ABP retention between CCI groups (**B**) in patients with RMD over six months estimated using Bayesian exponential regression adjusted for age, sex, and disease subtype. ABP: ABP501 biosimilar; CCI: Charlson Comorbidity Index; RMD: inflammatory rheumatic musculoskeletal diseases

Adjusting for age, sex, and disease subtype showed somewhat shorter retention rates for patients with CCI > 0 (Fig. 4A), but the distribution of the difference also includes 0 (estimate -0.0689, 95% CrI -0.2246 to 0.0234, Fig. 4B).

## Discussion

In this retrospective study, we successfully analysed the retention rates and reasons for discontinuation of the bsDMARD ABP over 6 months in patients with inflammatory RMD and investigated the role of comorbidity as a potential risk factor for treatment discontinuation. Almost 40% of patients had a CCI score > 0. As many as 75% of patients continued treatment with bsDMARD ABP over 6 months after a systematic non-medical switch from originator ADA. However, in this study no association between comorbidity as assessed by CCI and ABP retention rates could be shown.

To date, only few observational studies on the use of the bsDMARD ABP in patients with inflammatory RMD have been published. The 6-month ABP retention rate of 75% in the present study is somewhat lower than the 18-month ABP retention rate of 88% recently reported in a similar real-life study [18]. A reason could be the inclusion of switches at a timepoint earlier than October 2018. The year of treatment start has been shown to have a negative effect on retention rates, which is probably related to the increasing number of treatments available over time [18].

Patients with treatment persistence were older and had a longer disease duration than the 25% of patients who stopped treatment with ABP within 6 months. These patients mostly switched to another ADA bsDMARD (11%) or back to originator ADA (9%). Only a few patients switched to a different bDMARD or dropped out (3% each). The main reason for switches were adverse events, mostly rather subjective complaints. Of interest, patients who switched back to originator ADA had the longest treatment duration with the originator ADA (49 months) before the switch compared to the other groups. This suggests that a long duration of treatment with originator ADA could be considered as an indicator of effectiveness—with a greater likelihood of experiencing adverse effects after switching to a bsDMARD. In these patients, a back switch to originator ADA may have a good chance to be effective.

Data on switching from originator ADA to bsDMARD ABP in patients with inflammatory RMD are scarce, and the role of comorbidities in this context has not been studied to date. Thus, to the best of our knowledge, this is the first study to evaluate comorbidity as a risk factor for discontinuation of the bsDMARD ABP after non-medical switching from originator ADA in patients with inflammatory RMD. The most prevalent comorbidities were cardiovascular (40%) followed by skin (33%), gastroenterological (20%) and eye symptoms and diseases (20%). There was no evidence of impaired retention to ADA bsDMARD in a non-medical switch scenario with a follow up period of 6 months in RMD patients with comorbidities compared to those without. However, only 38% of our patients had a CCI > 0 – indicating a relatively healthy and/or well treated cohort.

Our study has some limitations. The main limitation is the observational retrospective study design. However, because non-medical switch scenarios in a real-world setting depend on economic decisions, application of a prospective study design is not feasible. Despite adjusting for confounding, we can, for example, not exclude that patients with less severe courses of disease were preferentially switched. The second limitation is the relatively low number of participants. However, a Bayesian approach was used, obtaining estimates that are valid for any sample size. Third, due to the retrospective design of our study no comparator group of patients with no change in medication could be studied because the switch was mandatory for all patients on originator adalimumab. Finally, we did not measure antidrug antibody against adalimumab as a possible reason for treatment discontinuation in our cohort. A cohort study on originator ADA showed that patients without anti-adalimumab antibodies had the best outcomes, and adalimumab-treated patients with anti-adalimumab antibodies the worst [19]. In a recent systematic review, it was shown that immunogenicity rates of originator ADA averaged 24.9% across studies and varied significantly over time [20]. Of note, an increase over time in the reported immunogenicity rates was detected in this systematic review. Time dependency may also have influenced our analysis. Immunogenicity data of bsDMARD ABP are scarce [21, 22]. However, in an open-label extension study 18.2% of subjects developed binding antidrug antibodies and 6.9% developed neutralizing antidrug antibodies. The rates of antidrug antibodies formation were similar between subjects who transitioned from adalimumab and those who continued on ABP 501 [23].

## Conclusion

Disease activity and physical function tended to be worse among patients with inflammatory RMD and comorbidities. No evidence of impaired retention to ADA bsDMARD in a non-medical switch scenario with a follow up period of 6 months in RMD patients with comorbidities compared to those without was found.

## Supplementary Information

Supplementary Material 1.

## Data Availability

Data are available upon reasonable request.

## Abbreviations

ABP: ABP501 biosimilar
ADA: Adalimumab
ASDAS: Ankylosing Spondylitis Disease Activity Score
axSpA: Axial spondyloarthritis
BASDAI: Bath Ankylosing Spondylitis Disease Activity Index
BASFI: Bath Ankylosing Spondylitis Functional Index
bDMARDs: Biologic disease modifying drugs
bsDMARDs: Biosimilar disease modifying drugs
BSRBR-AS: British Society for Rheumatology Biologics Register in Ankylosing Spondylitis
CCI: Charlson Comorbidity Index
CrI: Credible interval
CRP: C-reactive protein
DAS28: 28-Joint Disease Activity Score
FFbH: Funktionsfragebogen Hannover
HAQ: Health Assessment Questionnaire
HR: Hazard ratio
IBD: Inflammatory bowel disease
IQR: Interquartile range
PRO: Patient-reported outcomes
PsA: Psoriatic arthritis
RA: Rheumatoid arthritis
RMD: Rheumatic musculoskeletal diseases
SD: Standard deviation
