# Supplementary material for: A Bayesian model to analyse the association of comorbidities with biosimilar treatment retention in a non-medical switch scenario in patients with inflammatory rheumatic musculoskeletal diseases
Source: Arthritis Res Ther. 2024 Sep 4;26:155. doi: 10.1186/s13075-024-03386-7 (PMC11373462; doi:10.1186/s13075-024-03386-7)
Supplement: Supplementary file 1 — Supplementary Material 1. [file 13075_2024_3386_MOESM1_ESM.docx]

**Supplementary Table 1 Patients and disease characteristics at baseline (time of non-medical switch) stratified by outcome after 6 months**

| **Characteristic** | **Outcome after 6 months** | | | | |
| --- | --- | --- | --- | --- | --- |
|  | **Continuation bsDMARD ABP, N = 83^1^** | **Switch to other bsDMARD ADA, N = 12^1^** | **Back-switch to originator ADA, N = 8^1^** | **Switch to other bDMARD, N = 4^1^** | **Drop out, N = 4^1^** |
| Age (years) | 53 (13) | 44 (15) | 49 (19) | 42 (10) | 58 (27) |
| Male | 50 (60.2%) | 8 (66.7%) | 3 (37.5%) | 1 (25.0%) | 3 (75.0%) |
| Diagnosis |  |  |  |  |  |
| axSpA | 52 (62.7%) | 8 (66.7%) | 3 (37.5%) | 3 (75.0%) | 2 (50.0%) |
| RA | 17 (54.8%) | 1 (25.0%) | 2 (40.0%) | 1 (100.0%) | 2 (100.0%) |
| PsA | 11 (35.5%) | 2 (50.0%) | 2 (40.0%) | 0 (0.0%) | 0 (0.0%) |
| Other | 3 (9.7%) | 1 (25.0%) | 1 (20.0%) | 0 (0.0%) | 0 (0.0%) |
| Disease duration (years) | 6.8 (7.2) | 4.9 (4.2) | 4.0 (1.7) | 1.5 (0.6) | 3.8 (3.4) |
| CRP, mg/dl, median (IQR) | 0.10 (0.10, 0.30) | 0.10 (0.10, 0.30) | 0.10 (0.10, 0.30) | 0.10 (0.10, 0.30) | 0.10 (0.10, 0.30) |
| Duration originator ADA therapy (month) | 44 (28) | 30 (25) | 49 (22) | 11 (10) | 22 (19) |
| Number of previous csDMARD therapies | 1.07 (1.00) | 0.83 (0.72) | 1.25 (1.16) | 0.25 (0.50) | 0.50 (0.58) |
| Number of previous bDMARD therapies | 1.54 (0.83) | 1.50 (0.67) | 1.63 (0.74) | 1.25 (0.50) | 1.00 (0.00) |
| Current csDMARDs intake | 35 (42.2%) | 3 (25.0%) | 3 (37.5%) | 1 (25.0%) | 1 (25.0%) |
| Current NSAIDs intake | 13 (15.7%) | 2 (16.7%) | 0 (0.0%) | 0 (0.0%) | 1 (25.0%) |
| Current glucocorticoids intake |  |  |  |  |  |
| none | 66 (80.5%) | 12 (100.0%) | 6 (75.0%) | 4 (100.0%) | 4 (100.0%) |
| <5 mg | 9 (11.0%) | 0 (0.0%) | 1 (12.5%) | 0 (0.0%) | 0 (0.0%) |
| 5-10mg | 7 (8.5%) | 0 (0.0%) | 1 (12.5%) | 0 (0.0%) | 0 (0.0%) |
| Charlson Comorbidity Index |  |  |  |  |  |
| =0 | 51 (61.4%) | 7 (58.3%) | 5 (62.5%) | 4 (100.0%) | 2 (50.0%) |
| >0 | 32 (38.6%) | 5 (41.7%) | 3 (37.5%) | 0 (0.0%) | 2 (50.0%) |
| Gastroenterological comorbidities | 15 (18.1%) | 3 (25.0%) | 2 (25.0%) | 2 (50.0%) | 0 (0.0%) |
| Inflammatory bowel disease | 4 (4.8%) | 0 (0%) | 2 (25%) | 1 (25%) | 0 (0%) |
| Hepatic comorbidities | 6 (7.2%) | 1 (8.3%) | 1 (12.5%) | 0 (0.0%) | 0 (0.0%) |
| Hematological conditions | 5 (6.0%) | 1 (8.3%) | 0 (0.0%) | 0 (0.0%) | 0 (0.0%) |
| Cardiovascular comorbidities | 35 (42.2%) | 1 (8.3%) | 4 (50.0%) | 1 (25.0%) | 3 (75.0%) |
| Neurological and psychological comorbidities | 17 (20.5%) | 1 (8.3%) | 0 (0.0%) | 0 (0.0%) | 1 (25.0%) |
| Metabolic comorbidities | 10 (12.0%) | 2 (16.7%) | 2 (25.0%) | 0 (0.0%) | 2 (50.0%) |
| Osteoporosis | 19 (22.9%) | 0 (0.0%) | 1 (12.5%) | 0 (0.0%) | 0 (0.0%) |
| Lung diseases | 11 (13.3%) | 0 (0.0%) | 1 (12.5%) | 0 (0.0%) | 1 (25.0%) |
| Skin diseases | 27 (32.5%) | 4 (33.3%) | 3 (37.5%) | 2 (50.0%) | 1 (25.0%) |
| Psoriasis | 17 (20%) | 2 (17%) | 1 (12%) | 1 (25%) | 0 (0%) |
| Eye diseases | 19 (22.9%) | 2 (16.7%) | 1 (12.5%) | 0 (0.0%) | 0 (0.0%) |
| Uveitis | 8 (9.6%) | 1 (8.3%) | 1 (12%) | 0 (0%) | 0 (0%) |
| Kidney diseases | 10 (12.0%) | 0 (0.0%) | 1 (12.5%) | 0 (0.0%) | 1 (25.0%) |
| ^1^If not stated otherwise, data are reported as mean (SD) and n (%). ABP: ABP501 biosimilar; ADA: adalimumab; axSpA: axial spondyloarthritis; b/bs/csDMARD(s): biological/biosimilar/conventional synthetic disease-modifying anti-rheumatic drug(s); CRP: C-reactive protein; IQR: interquartile range; NSAID(s): nonsteroidal anti-inflammatory drug(s); PsA: psoriasis arthritis; RA: rheumatoid arthritis, SD: standard deviation. | | | | | |

**Supplementary Figure 1 Disease activity and physical function at baseline, month 3, and month 6**


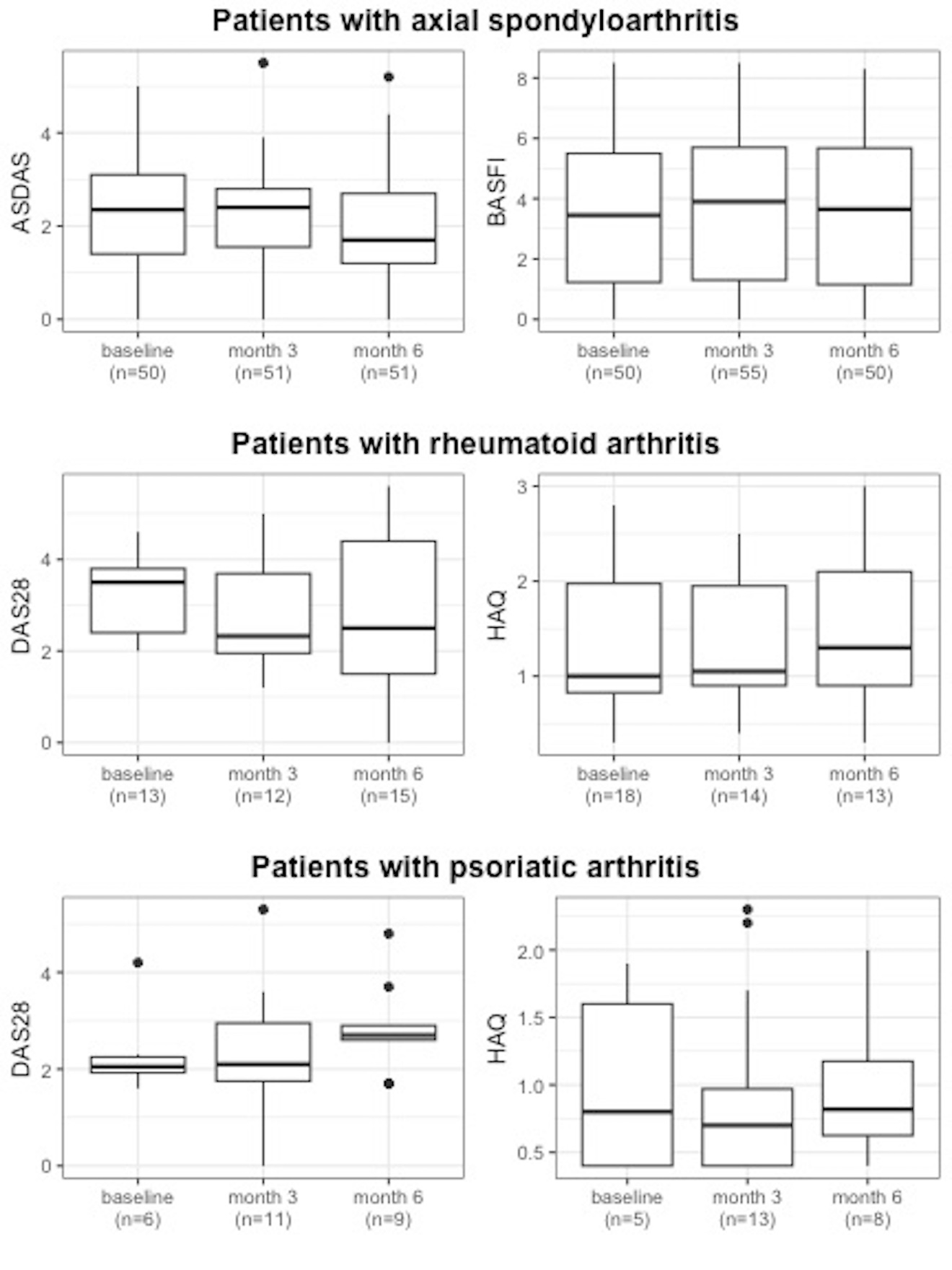


ASDAS: Ankylosing Spondylitis Disease Activity Score; BASFI: Bath Ankylosing Spondylitis Functional Index; DAS28: 28-joint Disease Activity Score; HAQ: Health Assessment Questionnaire.
